# Serum Vitamin D Level and Clinical Risk Factors for Benign Paroxysmal Positional Vertigo

**DOI:** 10.7759/cureus.99215

**Published:** 2025-12-14

**Authors:** Cesar A Nava-Gaytán, Hiram H Plata-Huerta, César A Ramos-Delgado, Jose Treviño-González

**Affiliations:** 1 Department of Otolaryngology, Head and Neck Surgery, Hospital Universitario “Dr. Jose E. Gonzalez" Universidad Autonoma De Nuevo Leon, Monterrey, MEX

**Keywords:** 25 (oh) vitamin d, benign paroxysmal positional vertigo, canalith repositioning, peripheral vertigo, vitamin d serum level

## Abstract

Objective: To identify the most prevalent comorbidities and the serum 25-hydroxyvitamin D levels that affect the onset and recurrence of benign paroxysmal positional vertigo.

Study design: This is a prospective cross-sectional descriptive study.

Setting: The study was conducted at Hospital Universitario "Jose Eleuterio Gonzalez" in Monterrey, Mexico.

Methods: A total of 117 patients over 18 years old who came for the first time to consultation complaining of dizziness and/or vertigo compatible with benign paroxysmal positional vertigo were included. Patients with positive vertiginous symptoms evoked with diagnostic test maneuvers were treated. A complete medical history was registered, the Dizziness Handicap Inventory was applied, and 25-hydroxyvitamin D serum levels were measured.

Results: From a total of 70 patients, the most prevalent comorbidities present were arterial hypertension (37.6%) and type 2 diabetes mellitus (29.1%). The most common cause of benign paroxysmal positional vertigo was idiopathic (67.5%), while in the secondary group, sudden neurosensorial hearing loss was the most common condition (6%). The mean average serum 25-hydroxyvitamin D level was 23.07 ng/ml, and insufficiency (42.9%) was the most common serum level present in the population of the study.

Conclusions: The comorbidities identified in this study, along with the deficiency levels of 25-hydroxyvitamin D, were factors associated with BPPV in our cohort and may have contributed to the onset and recurrence of benign paroxysmal positional vertigo; thus, we suggest that prospective studies should address this possible association and, if so, treat it to accomplish an effective treatment.

## Introduction

Benign paroxysmal positional vertigo (BPPV) was first described in 1921 by Robert Bárány [1]. It is the most frequent peripheral vestibular end-organ disorder, with a cumulative incidence of 10% in the general population [2,3]. A German study in an adult population revealed a lifetime prevalence of 2.4%, with a one-year prevalence of 1.6% and an incidence of 0.6% per year [4,5]. It has been estimated that 9% of the elderly have unrecognized BPPV, and other recent studies have found BPPV to be present in 40% of geriatric patients, with an overall general prevalence of 3.4% in individuals aged >60 years [6].

Several comorbidities have been identified as risk factors for the development of BPPV, such as hypothyroidism, osteopenia/osteoporosis, and low serum vitamin D levels [6-8]. Secondary etiologies related to otologic diseases, such as Meniere's disease, sudden neurosensorial hearing loss (SNHL), vestibular neuritis, otologic surgery, head trauma, vestibular migraine, prolonged bed rest, and dental procedures, have also been described [1,8-10]. In addition, the specific pattern of affection in the semicircular canals, which might be unilateral, bilateral, multicanal, or recurrent, influences the effectiveness of canalith repositioning maneuvers (CRM) in the treatment of BPPV [11-13].

This study aimed to identify the most prevalent comorbidities and serum 25-hydroxyvitamin D (25 (OH) vitamin D) levels that may influence the onset and recurrence of BPPV.

## Materials and methods

A prospective cross-sectional study was performed between January 2022 and January 2024 at the Otorhinolaryngology and Head and Neck Surgery department of the Hospital Universitario “Jose Eleuterio Gonzalez,” a tertiary center in Monterrey, Mexico.

Patients over 18 years who came for the first time to consultation, complaining of dizziness and/or vertigo compatible with BPPV based on Bárány Society guidelines and positive diagnostic test maneuvers (Dix-Hallpike, McClure, and cephalic hyperextension) were included [1,3]. A total of 117 patients were included, defining positive diagnostic test maneuvers for patients with vertigo triggered by diagnostic maneuvers with or without characteristic nystagmus, to identify the proportion of subjective BPPV (S-BPPV). The exclusion criteria included patients younger than 18 years; those exhibiting clinical characteristics of central vertigo, such as neurological deficits, nystagmus features inconsistent with BPPV, or positive signs of central vertigo upon examination; prior use of vestibular sedatives within seven days before consultation; or current use of vitamin D supplementation.

All patients underwent a complete medical history, otoscopy examination, and vestibular exploration by a certified otolaryngologist. Patients presenting with vertiginous symptoms evoked by diagnostic test maneuvers with or without nystagmus were evaluated using the Dizziness Handicap Inventory (DHI) and treated with CRM, such as Epley, Lempert, and Semont, based on the corresponding stimulated semicircular canal. A follow-up appointment was scheduled for one week with a blood serum 25 (OH) vitamin D test.

During follow-up, the DHI was applied again to measure the effectiveness of the repositioning maneuvers. Diagnostic test maneuvers were repeated in patients with persistent symptoms, and if they were positive, they were retreated with CRM. Patients with levels lower than 30 ng/ml were supplemented with oral intake of 4000 IU of 25 (OH) vitamin D daily based on reviewed regimens [14]. Based on the Endocrinology Society classification, levels are defined as normal (30-100 ng/ml), insufficiency (20-29 ng/ml), deficiency (12-19 ng/ml), and severe deficiency (lower than or equal to 11 ng/ml). As part of a two-year protocol, each patient was monitored for a minimum duration of three months following the initial oral administration of the supplement, with a focus on self-reported recurrence of vestibular symptoms.

We analyzed the demographics and comorbidities of the population, including the type, recurrence, laterality, semicircular canal affected, presence of multicanal affection, number of repositions needed, serum 25 (OH) vitamin D levels, DHI scores, seasonal incidence, and use of vestibular sedatives in patients diagnosed with BPPV.

Sample size calculation was conducted using the formula for estimating a proportion in a finite population to determine the proportion of vitamin D deficiency among patients with BPPV. The proportion of vitamin D deficiency was determined to be 85%, with a precision or magnitude of error of 5% (+/-0.5). The above, together with a Z value of 1.64 given by a significance of 0.05 and a power of 80%, required at least 59 study subjects. The calculation was performed based on the reported literature [15]. 

Data were reported using standard statistical methods. Continuous variables with normal distributions are presented as means with standard deviations, whereas those with non-normal distributions (based on the Kolmogorov-Smirnov test) are presented as medians with interquartile ranges. Categorical variables are expressed as percentages. Comparisons of numerical data were performed using an unpaired t-test (parametric data) or Mann-Whitney U test (nonparametric data). When appropriate, comparisons of categorical data were made using the x² and Fisher’s exact tests. The odds ratio was calculated to estimate the risk of recurrence, the necessity of more than one repositioning treatment, and post-treatment DHI scale score reduction. Statistical significance was set at p < 0.05. The IBM Corp. Released 2024. IBM SPSS Statistics for Windows, Version 30. Armonk, NY: IBM Corp. statistical package (RRID:SCR_016479) was used for analysis.

The study was approved by the Universidad Autonoma de Nuevo Leon Institutional Review Board (OT24-00010) and conducted in accordance with ethical standards, the Regulations of the General Health Law on Health Research and the Declaration of Helsinki, and current international codes and standards of good clinical research practice.

## Results

All demographic data and clinical outcomes are described in Table 1. In this study, 117 patients were included; 92 patients (78.6%) were female, and 25 (21.4%) were male. The overall mean age was 58.50±12.5. The most prevalent comorbidities were arterial hypertension in 37.6% and T2DM in 29.1%. BPPV was more frequent during the winter season in 31% of patients.

**Table 1:** Demographic, Clinical, and Pathological Profiles, Including Semicircular Canal Involvement, Comorbidities, Seasonal Presentation, and DHI Scores. Summary of demographic data, clinical characteristics, otologic and non-otologic pathologies, semicircular canal involvement, comorbidities, seasonal presentation patterns, medication history, and Dizziness Handicap Inventory (DHI) scores of the study population. SD, Standard Deviation, BPPV: Benign paroxysmal positional vertigo.

Characteristics	BPPV patients
	(n=117)
Demographics	
Age-years (mean±SD)	58.50±12.5
Men	58.5±12.4
Women	58.5±12.8
Sex - N (%)	
Men	25 (21.4%)
Women	25 (21.4%)
Clinical	
Recurrence – N (%)	17 (13.7%)
Etiology - N (%)	
Idiopathic	79 (67.5%)
Subjective	9 (7.7%)
Secondary	38 (32.5%)
Ear pathology	21 (17.9%)
Sudden Neurosensorial Hearing Loss	7 (6%)
Meniere disease	6 (5.1%)
Vestibular neuritis	4 (3.4%)
Previous ear surgery	4 (3.4%)
Non ear pathology	17 (14.4%)
Head trauma	9 (7.7%)
Osteoporosis	6 (5.1%)
Prolonged bed rest	1 (.8%)
Dental surgery	1 (.8%)
Semicircular canal affected -N (%)	
Posterior	100 (86.1%)
Lateral	30 (25.8%)
Unilateral	101 (87.8%)
Multicanal	14 (12.2%)
Laterality (%)	
Right	63.30%
Left	55.60%
Comorbidities - N (%)	
Arterial hypertension	44 (37.6%)
Diabetes Mellitus type 2	34 (29.1%)
Hypothyroidism	26 (22.2%)
Allergic rhinitis	15 (12.7%)
Dyslipidemia	14 (11.8%)
Osteopenia	6 (5.1%)
Osteoporosis	6 (5.1%)
Heart disease	8 (6.8%)
Asthma	7 (5.7%)
Season prevalence – N (%)	
Winter	36 (31%)
Auttum	28 (24.1%)
Summer	27 (23.3%)
Spring	26 (22.3%)
Vestibular sedative drugs – N (%)	40 (34.2%)
Diphenidol	22 (18.8%)
Betahistine	15 (12.8%)
Cinarizine	3 (2.6%)
Dizziness Handicap Inventory score	
Pre-treatment	62 (38-78.5)
Post-treatment	20 (4-48)
Thyroid level (mean, SD)	
TSH (mIU/dL)	2.53±2.6
free T4 (ng/dL)	1.39±0.2
free T3 (pg/mL)	2.41±0.8

The most common cause of BPPV was idiopathic in 67.5% of patients; within the idiopathic group, 7.7% of patients were categorized with S-BPPV, who did not present characteristic nystagmus but did present vertiginous symptoms with diagnostic maneuvers. Secondary BPPV was present in 32.5% of patients, with SNHL being the most prevalent antecedent of ear pathology (6%). In contrast, head trauma was the most prevalent condition (9%) in non-ear pathology among patients with BPPV.

The posterior semicircular canal was the most affected, at 86%, followed by the horizontal canal at 25%. However, no involvement of the anterior canal was observed. The right side was the most frequently affected in 63.3%. Of the 117 patients, 87.8% had single-canal involvement, in contrast to the 12.2% who had multicanal involvement. During the two years that the study was conducted, 13.7% of patients returned with recurrent symptoms, and recurrent BPPV was diagnosed.

The association between the risk of recurrence and the necessity of more than one repositioning with a CRM was estimated for the comorbidities, pattern of affection, and etiology of secondary BPPV. The risk of recurrence association was estimated for the antecedent of hypertension (OR .09 [95% CI, 0.01-0.7]), showing a protective effect. The presence of T2DM (OR 2.79 [95% CI, 1.08-7.17]) and multicanal involvement (OR 3.7 [95% CI, 1.1-12]) increased the risk of requiring more than one repositioning treatment in a single ear. The remaining variables showed no statistically significant effects.

Seventy patients underwent 25 (OH) vitamin D testing. The overall mean level was 23.07±8.1 ng/ml. The most common levels were insufficiency and deficiency, at 42.9% and 32.9%, respectively. Multiple comparisons of vitamin D levels were performed; idiopathic versus secondary and recurrent versus non-recurrent BPPV did not show statistical significance (p >.05). Patients with dyslipidemia presented higher levels of 25 (OH) vitamin D than patients without the condition (28.6±9 vs. 22.15±7.6; p=.01). In addition, patients who needed more than one repositioning with CRM, those who presented recurrence, primary BPPV, multicanal involvement, and those whose DHI score improved by less than 50% after repositioning had lower levels of vitamin D than those without these conditions, although this was not statistically significant (p >.05). 25 (OH) vitamin D results are shown in Table 2. 

**Table 2:** Results on Categories of Serum Vitamin D. Serum 25-hydroxyvitamin D levels were classified as deficient (<20 ng/mL), insufficient (20–30 ng/mL), or sufficient (>30 ng/mL), and a description of the subtype of semicircular canal was included.

25-hydroxyvitamin D mean average (ng/ml)	23.07 ± 8.1
25-hydroxyvitamin D levels clasification	N(%)
Normal	14 (20%)
Insufficient	30 (42.9%)
Deficiency	23 (32.9%)
Severe deficiency	3 (4.3%)

Concerning the DHI, the pretreatment scale score significantly decreased after CRM, from 62 (38-78.5) to 20 (4-48), with a statistical significance of p<.001. A total of 34.2% of patients reported a history of utilizing vestibular suppressant medications for the symptomatic relief of dizziness and/or vertigo, with diphenidol being the most used in 18.8% of the patients.

During data recollection, no harm or adverse effects were found or reported by the patients.

## Discussion

According to the reviewed literature, this is the first study in the northeast Mexican population describing the demographics, clinical outcomes, and serum 25 (OH) vitamin D levels in patients with BPPV. We focused on the clinical comorbidities, etiologies of BPPV, pattern of affected canals, and serum 25 (OH) vitamin D levels, and how they affect the treatment outcome in BPPV and as risk factors for recurrent BPPV. In agreement with prior studies, the demographic and clinical characteristics of BPPV in this study are congruent with the reported literature [2,6,16]. Female predisposition for BPPV is considered a risk factor for recurrence compared with male gender [9].

The most prevalent comorbidities in our BPPV population were arterial hypertension and T2DM. Along with osteoporosis and dyslipidemia, these comorbidities are in accordance with the reported literature by Pecci et al. regarding the prevalence of comorbidities among patients with BPPV [17]. Cheng J. et al., in their systematic review and meta-analysis, identified the risk factors for recurrent BPPV. They found that T2DM and dyslipidemia, which were present in 29.1% and 11.8% of patients, respectively, are risk factors for recurrence based on this study [9].

The unexpected protective effect of hypertension against BPPV recurrence (OR 0.09 [95% CI, 0.01-0.7]) warrants careful interpretation. These findings contrast with the results of Cheng J et al. and De Stefano A et al., who identified hypertension as a risk factor for recurrence and an increased risk of recurrence of hypertension when associated with T2DM, respectively. Given our limited sample size and the wide confidence interval approaching zero, this result may represent a misleading finding rather than a true protective mechanism. Alternatively, adequate antihypertensive treatment could theoretically reduce otolithic instability; however, this hypothesis requires validation in larger controlled studies. In contrast, the association between T2DM and an increased risk of requiring multiple repositioning treatments (OR 2.79 [95% CI, 1.08-7.17]) aligns with the existing literature and represents a clinically significant finding [9-18]. Arterial hypertension, present in 37.6% of our patients, showed a protective effect when associated with recurrence.

Hypothyroidism history was present in 22.2% of patients in this study (Table 1), and it has been described in different studies as a risk factor for recurrent BPPV. In accordance with this prevalence, Papi et al., in a multicenter control study, showed that hypothyroidism was present in 21% of patients with BPPV [19]. Power et al. showed that hypothyroidism was present in 17.6% of patients with BPPV in their retrospective analysis [20]. Also, L. Tricarico et al. demonstrated that hypothyroidism on hormonal replacement therapy and autoimmune chronic thyroiditis with positive thyroglobulin and thyroid peroxidase antibodies are risk factors for recurrent BPPV [7,9]. The occurrence of hypothyroidism within this cohort was observed as an association; therefore, extended follow-up studies involving control groups and adjusted confounders are necessary to evaluate this as a potential risk factor.

As described in the literature, idiopathic BPPV is the most frequently reported etiology, with 50-70%; in our study, idiopathic BPPV was present in 67.5%, in congruence with the literature [10-21]. We recovered the prevalence of all the described secondary etiologies in our BPPV population to set a prevalence and compare it with that described in the literature. Secondary BPPV was present in 32.5% of patients based on prior ear and non-ear pathology. SSNHL was the most frequent previous ear pathology, at 6%.

About Meniere's disease, different prevalences have been described; Karlberg and colleagues reported a .5% prevalence, Hugh Ca et al. reported a bigger prevalence of 31%, and Groos et al. reported a 5.5% prevalence. In our study population, Meniere's disease was present in 5.1%, similar to what Gross reported [10]. Cheng et al. also studied the association of Meniere´s disease with recurrent BPPV with no correlation, so it was not considered a risk factor for recurrence [7,9,22]. Baloh et al. and Perez et al. reported the prevalence of vestibular neuritis of 17% and 15%, respectively, which is different from our results, which reported a prevalence of 3.4%. Considering that the annual incidence of vestibular neuritis is approximately 3.5% to 15.5% per 100,000 persons and approximately 4% to 9.8% of adult patients, we consider that the proportion of patients with BPPV and the antecedent of vestibular neuritis should be lower than that reported in the described studies. BPPV related to previous ear surgeries, Atacan et al. reported a prevalence of 6% BPPV secondary to stapedectomy [10,21,23,24]. We report a prevalence of 3.4% associated with mastoidectomy procedures. 

In secondary BPPV related to non-otologic etiologies, the most common cause described in the literature is head trauma, with a prevalence of 7-17% [10]. Different studies support this prevalence range. Teggi et al., in a sample of 3042 patients with recurrent BPPV, found a prevalence of 16.9% associated with head trauma [12]. Perez and colleagues described the onset of BPPV from different etiologies, reporting the prevalence of head trauma of 9% [21]. Motin et al. studied 150 patients with severe head trauma, among whom 6.6% were diagnosed with BPPV [25]. Similarly, we found a prevalence of 7.7%, with head trauma being the most frequent etiology of secondary BPPV. Although head trauma has been described as the main cause of recurrence in BPPV in different studies, Cheng et al. found in their meta-analysis that it is not a risk factor for recurrence [9].

Osteoporosis, osteopenia, and bone mineral density disorders are pathologies related to aging in women. Pecci et al. reported osteoporosis as a prevalent comorbidity in patients with BPPV in 23% [17]. Among women with BPPV, Vibert et al. found that 75% of women with BPPV had osteopenia or osteoporosis [26]. In our study, we found an equal prevalence of osteopenia and osteoporosis, at 5.1%. Wang Z et al. described a prevalence of 39.8% and 29.1% of osteopenia and osteoporosis, respectively, among a population of 103 women with recurrent BPPV. They concluded that these disorders may influence recurrence. This idea is supported by Chen et al., who found osteoporosis to be a risk factor for recurrence [8,9].

In our study, we just had one patient with BPPV related to prolonged bed rest. We report the case of a 28-year-old woman with no comorbidities and previously healthy left submandibular and sublingual sialadenitis that required six days of intravenous treatment in the hospital. She developed a posterior canal BPPV during her hospitalization; the patient spent all day and night lying in bed.

Multicanal BPPV has been described as a rare clinical entity; however, a previous study reported a prevalence between 4.7% and 12.2%, and it is more frequent in post-traumatic BPPV [10,20]. In our study, multicanal BPPV was present in 12.2% of a population of 117 patients, which is consistent with the literature. Shim DB et al. showed a prevalence of 4.6% among 1054 patients; according to Soto-Varela A. and colleagues in their study, the prevalence was about 8% in a population of 583 patients, and Balatsaorus DG et al. reported a prevalence of 9.3% in his study of 345 patients [27,28].

In our study, we used the DHI scale to evaluate the success of CRM based on the persistence of symptoms referred to by the patients at the follow-up visit. We found that patients whose scores decreased by at least 50% had successful treatment that was clinically significant. Thus, we consider the DHI scale a useful, easy, fast, and cheap tool that is clinically sensitive for the follow-up of patients, especially for patients who have risk factors for recurrence or are affected by multicanal patterns and might need more than one CRM.

Low levels of 25 (OH) vitamin D, specifically deficiency, have been described by different studies, systematic reviews, and meta-analyses as an independent risk factor for recurrence in patients with BPPB. In addition, supplementation of insufficiency and deficiency levels is effective in decreasing the recurrence and intensity of BPPV episodes [17].

In our study, the mean serum 25 (OH) vitamin D level was 23.07 ng/ml, and the deficiency level was 32.9%. Only two studies had the same results related to average vitamin D level, whereas the concentration levels, the most prevalent, were insufficient in 42.7% and deficient in 32.9%, contrasting with the previous studies, which reported a deficiency with a prevalence greater than 40%. No significant difference was observed between the initial episode and the recurrence of BPPV. Even though it was not clinically significant, we suspect low levels of 25 (OH) vitamin D in patients who needed more than one repositioning with CRM, patients with recurrence, idiopathic BPPV, multicanal involvement, and those whose DHI scale score improved by less than 50% after the CRM [29].

The association of BPPV and seasonal incidence, and its correlation with vitamin D, was described by Shu L et al. They described in their results that during winter, patient numbers were significantly higher, and in the winter group 25 (OH), vitamin D levels were lower with deficiency levels [22]. In our study, BPPV was more frequently diagnosed during the winter season (31%).

The most described and accepted ratio of recurrence of BPPV is 50% in 10 years, the largest part (80%) of which occurred within the first year, based on a retrospective study by Brandt T. et al. [30]. Different reports have described different ratios of recurrence related to short-term recurrence. In our study, recurrence was observed in 13.7% of patients within two years. Based on current data from different studies, the percentage range for short-term recurrence after the first year is about 15-27% [21]. Different rates of recurrence might be within this range and even higher. They may differ according to the comorbidities and serum vitamin D concentrations present in each population studied, which were influenced by ethnicity and geographic location. We suggest that it is important to assess effectively the presence of comorbidities among BPPV patients because of its association with recurrence as described by De Stefano A et al., who described an odds ratio (OR) of 2.25 in the presence of one comorbidity and an OR of 2.84 in the case of two or more diseases, and supplement with 25 (OH) vitamin D in all cases suspecting insufficiency and deficiency [18].

The main limitations of this study are the small population of patients with BPPV, the small number of 25 (OH) vitamin D serum tests, in conjunction with the absence of a control group. While many of our findings align with existing literature, some results diverge. Consequently, further controlled prospective studies incorporating long-term follow-up and vitamin D supplementation are warranted.

## Conclusions

The comorbidities identified in this non-control observational study, alongside the deficiency levels of 25-hydroxyvitamin D, may contribute to hypothesizing the risk factors influencing the onset and recurrence of BPPV. Consequently, we recommend that future prospective studies explore this potential association. These findings lay the groundwork for developing appropriate treatment strategies to ensure effective management of this condition.
